# “They Need to Know the Science, but We also Need to Listen”: Perspectives of Black Rural Postpartum Mothers’ Health Care Providers And Support Persons

**DOI:** 10.1089/heq.2024.0051

**Published:** 2024-08-29

**Authors:** Natalie Hernandez-Green, Morgan V. Davis, Kaitlyn Hernandez-Spalding, Merna S. Beshara, Oluyemi Farinu, Kennedy Lewis, Sherilyn Francis, LeThenia Joy Baker, Sherrell Byrd, Andrea Parker, Rasheeta Chandler

**Affiliations:** ^1^ Morehouse School of Medicine, Atlanta, Georgia, USA.; ^2^ Emory University, Atlanta, Georgia, USA.; ^3^ Georgia Institute of Technology, Atlanta, Georgia, USA.; ^4^ Wellstar West Georgia Medical Center, LaGrange, Georgia, USA.; ^5^ SOWEGA Rising Coalition, Albany, Georgia, USA.

**Keywords:** mHealth, postpartum, Black women, maternal health disparities, rural health, mental health

## Abstract

**Background:**

In the United States, Black women are three times more likely to be affected by maternal mortality than White women. People who live in rural areas also face an increased risk. The objective of this study was to explore the perspectives of Black postpartum women’s support persons and health care providers, and the impact of race and rurality on their roles, to inform the development of a mobile health (mHealth) application focused on postpartum transitional care for rural Black women.

**Methods:**

Utilizing a semistructured designed discussion guide, we conducted four focus groups between July 2021 and October 2021. We asked support persons and health care providers to share their opinions about (1) postpartum needs, (2) the current hospital discharge process, (3) gender discrimination and experiences of racism and classism, and (4) suggestions for mobile application development.

**Results:**

Ten health care providers and seven support persons participated in the focus groups. A total of 57.1% of support persons identified themselves as a family member of the mother. In addition, 60% of health care providers indicated they practiced in a rural area at the time. Identified themes included race and rurality, emotional health, participants’ roles in relation to mothers’ needs, and the importance of technology for accessing information and resources.

**Conclusion:**

When describing their personal experiences, participants emphasized the importance of mHealth technology for helping Black postpartum mothers access health information. Insight from support persons and health care providers highlighted the challenges Black rural mothers face during the postpartum period and how our mobile application can be best utilized to address their needs.

## Introduction 

The maternal mortality crisis in the United States impacts Black women disproportionately. Rates for maternal mortality are about three times higher for Black women than White women.^1^ In addition, people who live in rural areas are 9% more likely to be affected by severe maternal mortality and morbidity.^2^ While the importance of a support system for pregnant people is highlighted in current literature, it is imperative to understand how race and rurality impact the support persons themselves.^3^ This study investigates how race and rurality can impact support persons’ and health care providers’ roles, and how their unique perspectives can inform a mobile application.

## Background

### Race and Rurality as a Support Person and Healthcare Provider

Compared with every other developed country, the United States ranks the highest for rates of maternal mortality.^3^ Residents of rural counties also face an increased risk for poor maternal health outcomes. More than half of all rural counties have no hospital-based obstetrical services.^2^ In Georgia, 93 rural counties have no hospital with a labor and delivery unit. Lack of access to these necessary services results in several barriers for mothers, including finding transportation, reaching care in a timely manner, and establishing a continuity of care.^4^

Rural women of color are at an even higher risk for poor maternal health outcomes. According to Hostetter et al, rural counties with a greater percentage of non-Hispanic Black women are more likely to lose access to obstetric services.^2^ In addition, Black women in rural Georgia are twice as likely to die from pregnancy-related causes than their White counterparts.^4^

The barriers and burdens rural Black women in Georgia face can impact the support persons/partners and health care providers who serve this population. For example, some partners or support persons may have to assist mothers in providing transportation. Furthermore, the closure of hospitals in rural areas has been shown to contribute to health care professional shortages in several specialties.^5^

### Emotional Health of Mother and Support Person

Poor mental health is one of the leading causes of maternal mortality in the world.^6^ One in 7 women in the United States will develop a perinatal mood disorder, which contributes to pregnancy-related and childbirth complications.^7^ Forty percent of Black women will experience poor mental health during their pregnancy and are more susceptible to perinatal mood disorders than White women.^8^ Moreover, the impact of poor mental health for Black women is greater because they are less likely to pursue mental health treatment due to stigma, financial barriers, and a lack of access to services.^8^ Maternal mental health conditions also affect the support persons who assist the mother with her motherhood journey. Support people can become distressed or emotional when a mother battling poor mental health becomes combative or difficult.^9^ However, support people have a crucial role in ensuring that the mother remains calm and has the most stress-free birthing process possible.

### Mothers’ Physical Health and Support Persons and Healthcare Providers Role

Women experience a multitude of mental, physical, and emotional stressors during the postpartum period. Studies show that mothers who receive “emotional, tangible, and informational support,” from a support person, family member, or provider are less likely to experience childbirth and postpartum complications.^10,11^ Likewise, mental health has been shown to be significantly affected by the emotional and practical help a mother receives from a partner or family member.^10^

Due to perceived racism and cultural beliefs, Black mothers are less likely to voice their needs to a health care provider.^12^ This leads to a barrier in communication, limits informational support, and significantly impacts the quality-of-care mothers receive during their pregnancy, delivery, and postpartum periods.

While attempts to improve mental health efforts for mothers during the postpartum period have been made, little is known about the expectations and mobilization of support for Black mothers in rural and underserved communities. A lack of support from a partner, family member, and even health care provider largely determines the fate of a mother’s postpartum period. Identifying the needs of Black mothers is crucial to their postpartum recovery and their ability to bond with and care for their newborns.

### Need for Technology to Access Information and Resources

Mobile phones and technology have become increasingly popular as a source of health information. Mobile health (mHealth) has the potential to provide a wealth of access to resources in communities where direct care from providers is limited. Many women in the postpartum period report using the internet and mHealth applications to obtain health-related information.^13–17^

Health care providers suggest that technology can help connect them with their patients during postpartum care, a transitional time, which should be an ongoing priority rather than a single encounter, with services and support tailored to each woman’s individual needs. In addition, the American College of Obstetricians and Gynecologists has highlighted the use of technology and mobile-app-based tools for optimizing postpartum care within and outside the clinic in their recent guidelines.^18^

Although the importance of support systems for pregnant and postpartum people is noted in current literature, the available discourse on the perspectives of support persons regarding the use of mHealth during the postpartum period is severely limited. Thus, this emphasizes the need for research elucidating support persons’ views on mHealth apps providing postpartum content to rural Black women as well as their support persons. Innovations in mHealth technology have the potential to be customized to better meet the needs of Black women and those with limited access to resources, support, and communication. The PM^3^ (Preventing Maternal Mortality Using Mobile Technology) mobile application was created to assist postpartum, rural Black women with health management and provide them with social and community resources. The versatility inherent in digital platform modalities represents a plethora of opportunities for education, prevention, and early symptom identification, as well as the facilitation of numerous treatment options.

## Frameworks

### Sojourner Syndrome

The Sojourner Syndrome theoretical framework was utilized in this study to address the intersectionality of race, gender, and class and to understand how each factor interacts to impact health outcomes for Black women. Furthermore, the Sojourner Syndrome framework focuses on the roles and identities Black women have adopted in the face of oppression. We utilized this framework to consider how institutional racism, sexism, and classism interact with Black women’s lived experiences to contribute to disparities in maternal morbidity rates, the quality of care and level of support they receive from support persons and providers, and negative impacts on postpartum mental health.^19^ From this framework, we were able to develop an interview guide that centers Black women and their experiences to understand how support persons and health care providers show up for mothers and to inform the development of the PM^3^ mHealth application.

### Maternal Mortality and Morbidity Measurement Framework

The Maternal Morbidity Measurement (MMM) Framework was developed in 2012 by the World Health Organization (WHO). This framework was designed to create a standardized definition, conceptualization, and assessment of maternal morbidity. In this study, we utilized the MMM framework to examine how policy, health systems, socioeconomic status, and preexisting health conditions collaborate to affect women’s reproductive health. Concluding the development of this framework, researchers emphasized the necessity of continuing primary care beyond the six-week postpartum period.^20^

## Methods

This study was conducted to understand the facilitators and barriers to postpartum health in rural Black mothers. Approval from the Morehouse School of Medicine Institutional Review Board was obtained before beginning the study. The research design, data collection, and analysis were conducted by a team of researchers who identify as people of color, have training in public health, and have experience working with Black postpartum women/postpartum women of color in community-based clinical settings. Throughout the study, we reflected on the analytic process as a group to arrive at consensus through investigator triangulation. We used the Standards for Reporting Qualitative Research (SRQR) as guidance for writing the current article. The SRQR reporting checklist is provided in Supporting Information: Appendix.

Focus groups were conducted with support persons of Black, rural mothers and health care providers to inform the production of a mobile application and postpartum continuum of care model. In addition, separate focus groups were conducted with Black, rural postpartum mothers themselves, published elsewhere.^21^ While the previously mentioned article centers Black rural postpartum mothers, our focus in this article is on the perspectives of support persons and health care providers. The theoretical and methodological approaches are similar for the article published on the Black mothers’ focus groups; however, the focus differs from that of this article published on support persons and health care providers. Support person study participants were eligible if they were identified by a mother as a support person (e.g., father, family member, doula, friend, peer) and/or if they provided support to a mother who had given birth within four weeks before recruitment. Health care providers were eligible if they identified as a physician, nurse practitioner, physician assistant, midwife, pediatrician, psychologist, doula, or lactation specialist. Providers were also considered eligible if more than 50% of their patient population were Black women and they spent more than 50% of their time in community-based settings (rural clinics, health departments, safety net clinics, etc.). To ensure the target population’s needs were represented in the research, a Community Advisory Board (CAB) of 12 people was established.

### Recruitment

The CAB consisted primarily of rural Black postpartum women (*n* = 6), representatives of community partner organizations (*n* = 3; SOWEGA Rising, Black Mammas Matter Alliance, Tri-County Rural Health), and health care providers (*n* = 3; WellStar West Georgia Medical Center, Georgia Department of Public Health, Georgia OBGYN Society). CAB members developed individual locally, culturally, and contextually appropriate noncoercive recruitment and enrollment processes to recruit support persons and providers into the study.^21^ This included providing information to participants at community events, supplying flyers, and providing contact information for participants to learn about the project and enroll. We also recruited participants using flyers and word of mouth.

### Survey and Data Collection

Focus groups with support persons and health care providers were conducted from July 2021 through October 2021. All participants were instructed to complete an eligibility survey before participating in the focus groups. After participants were deemed eligible, they completed a self-administered survey containing sociodemographic questions and provided written consent before participating in a focus group. Each focus group was moderated by a member of the research team and was recorded through Zoom. After the completion of the focus groups, the recording was transcribed by the research team.

Focus group discussion guides followed a semistructured design, utilizing the Sojourner Syndrome framework and the Maternal Morbidity Measurement framework. Participants were asked about their role in supporting Black, rural women’s postpartum experiences and needs; perceptions of the standardized hospital discharge process; personal experiences of classism, racism, and gender discrimination; and their suggestions for features of the PM^3^ mobile application. All information provided by participants was kept anonymous and confidential.

### Data Analysis

After data were collected, each transcript was reviewed, and three separate codebooks were created for each type of focus group. Five research team members manually coded and verified the transcripts. Afterward, each transcript and codebook were uploaded to a qualitative data analysis software. Using Dedoose version 9, team members compared the data across each code and identified major themes within the data. Direct quotes were then pulled from the data for discussion.

## Results

**FIG. 1.**
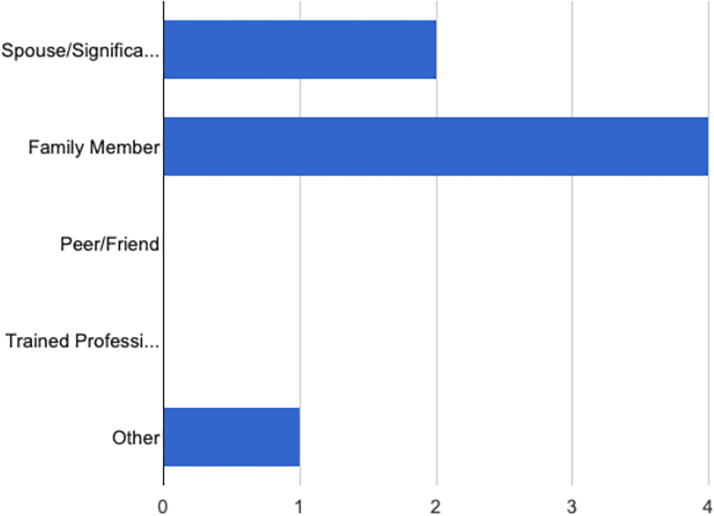
**“What is your relationship to the mother you support?”** Spouse/significant other (2, 28.6%), family member (4, 57.1%), peer/friend (0, 0.0%), trained professional (0, 0.0%), other (1, 14.3%).

**FIG. 2.**
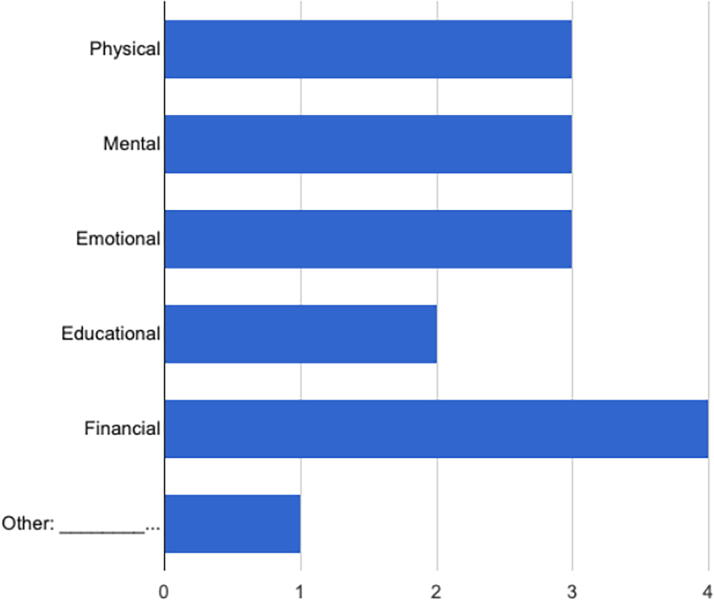
**“What type of support do you provide to the mother?”** Physical (3, 42.9%), mental (3, 42.9%), emotional (3, 42.9%), educational (2, 28.6%), financial (4, 57.1%), other: ___________________ (1, 14.3%).

A total of 10 support persons and 10 HCPs were screened in the study. Of the screened participants, 7 support persons and 10 HCPs participated in 4 focus group discussions (2 support person focus groups and 2 HCP focus groups). As depicted in Figures 1 and 2, most support persons identified as a spouse or family member and claimed to provide mental, physical, emotional, and financial support.

**FIG. 3.**
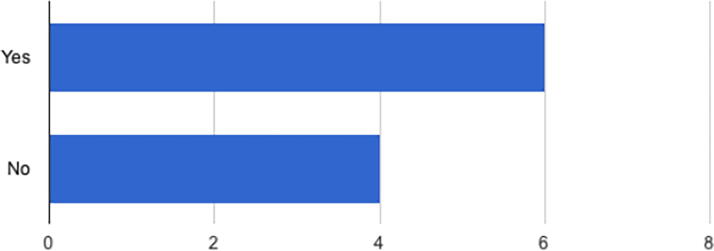
**“Is your practice in a rural location?”** Yes (6, 60.0%), no (4, 40.0%).

**FIG. 4.**
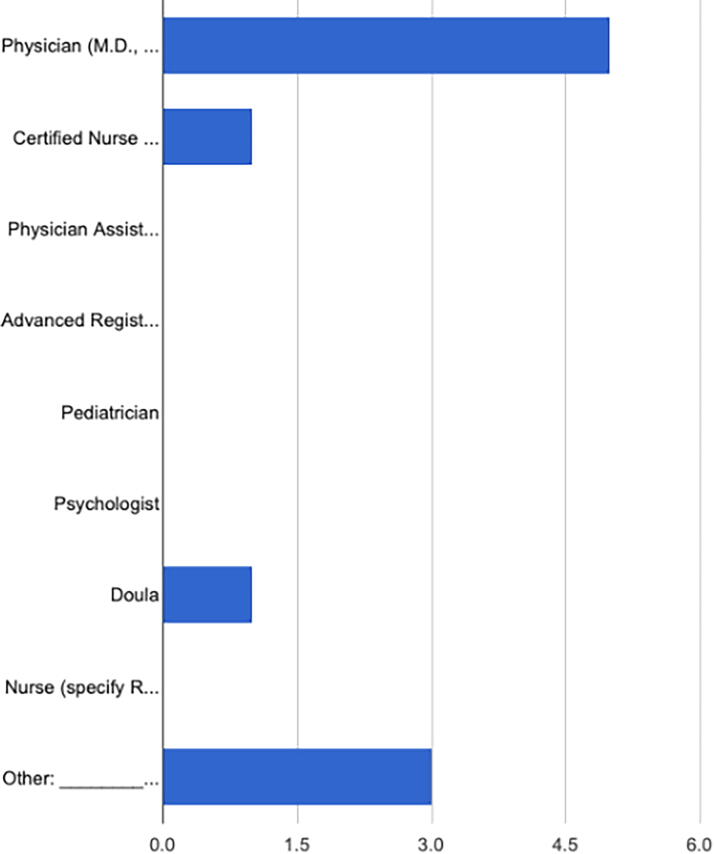
**“What is your healthcare profession?”** Physician (M.D., D.O.) (5, 50.0%), certified nurse midwife (CNM) (1, 10.0%), physician assistant (PA) (0, 0.0%), advanced registered nurse practitioner (ARNP) (0, 0.0%), pediatrician (0, 0.0%), psychologist (0, 0.0%), doula (1, 10.0%), nurse (specify RN, LPN, BSN) (0, 0.0%), other: ____ (3, 30.0%).

As shown in Figures 3 and 4, many of the health care providers practiced in rural areas and identified as physicians (*n* = 5). Other specialties included certified nurse-midwives (*n* = 1), doulas (*n* = 1), lactation consultants (*n* = 2), and breastfeeding peer counselors (*n* = 1).

### Race and Rurality as a Support Person and Health Care Provider

Support persons were asked about their perspectives as Black men living in rural Georgia. Mental health was a widely discussed topic; one participant stated, “Our whole system, itself, is built to break down the Black man.” He spoke about how the system creates trauma, and how this trauma impacts his role as a support person:

It creates trauma. We have to deal with all that trauma that we get from the system that’s created before we can properly love our wife, kids, anybody else. We have to deal with that trauma first. A lot of us in the Black community don’t deal with that trauma, because that’s looked down upon.

When asked about rurality, support persons discussed the challenges about living in the rural South. One support person described what it is like to live in a rural area and the necessity of having transportation:

When I see the structure that is constructed in a food desert, and in a foodscape, which is where we at right now and not having access to quality food. The closest grocery store here is about two, three miles, and they don’t have cars to get there. In rural Georgia, you need a car to get around.

When providers were asked about the effects of providing care to rural patients one shared,

When you live in a rural area, you don’t have access to the internet… I have one mom, who I have to call her and just like, let the phone ring, and then she actually has to drive somewhere, so she can call me back and we can talk…She doesn’t always have time to do that and so… we have our cell phones…but not everybody, when they’re sitting in their house actually has internet service. So, we’ve got to make allowances and try to reach everyone.

### Support Persons’ Experiences Caring for Black Mothers’ Emotional Health

The mothers’ support persons expressed, on several occasions, the importance of simply being present for the mothers during their postpartum journey and being attentive to the mother’s needs will improve her condition. One support person in particular argued one must ensure the mother’s physical and emotional needs are met:

The best way to go about helping a woman during postpartum to make sure that she is, uh, emotionally taken care of, you got to be there … you got to be there to hold her hand, you got to be there to help her out the bed, you got to be there, to just lay there sometimes next to her, giving her that emotional support. Now the physical support will come from, okay, I’m gonna wash the dishes, I’m gonna clean up you just lay down and rest.

Another common theme discussed within the support person focus groups was the tendency of Black Americans to avoid conversations about mental health and the hesitancy toward pursuing treatment for poor mental health. One support person felt these trends contribute to death among Black youth and Black parents must work to improve their own mental health to be the best version of themselves for their children. He stated:

Everyone says it is not talked about enough. We lose a lot of people to that discussion … I feel that parents need to be in a great mental state, even though they have their own challenges. I think being in a good state of mind, having good emotions behind everything, it will help the next generation in the long run.

### Health Care Providers’ Perspectives on Supporting Mothers’ Emotional Health

The main consensus among the health care providers who serve Black mothers was a lack of connectivity with their community, combined with a lack of resources, has negative implications for the mother’s postpartum experience. One provider mentioned how poor mental health affects a mother’s health journey and providers should support mothers, physically and emotionally, into their profession. She stated:

I think we have a responsibility to as a practice, to reach out to your people or somehow have a follow-up for them … So I think just being in tune with your, with your group with your practice.

Other providers also sought to provide emotional support to the mothers and described how important reliable support systems are for the mothers. The providers argued they must be open and transparent with their patients, so mothers feel comfortable pursuing follow-up care and asking questions. Notably, one provider mentioned how Black women often feel they must be their own problem-solvers and pushed back on this notion. She says:

I know culturally, we kind of faced that barrier, sometimes with Black women feeling like they have to do everything on their own, or that there’s no one else there to help them at baseline. I also think that a lot of moments that there are care gaps, just having someone there in the gap with the patient to help or having family or that added support makes a huge difference, especially when we’re talking about women who aren’t in the best resource-rich areas.

### Support Persons Perspectives on Supporting Mothers’ Health

Health care providers across focus groups provided their insight into a mother’s needs during the postpartum period. Across focus groups, several providers mentioned the necessity of informational and emotional support. One provider commented:

I think it’s two-sided. I think they need to know the science, but we also need to listen, because there’s so many stories of people, you’ve heard it from other patients who say, Well, I told them, I had this and they still sent me home.

Another provider mentioned the importance of mothers having a community, noting the informational advantages that are available to mothers who are surrounded by a community of women and various support people:

I’m saying community, but I was thinking friends, groups, they’re connected to it through those groups, they can get other resources and that’s what I like. I spoke to a lactation group a few weeks ago, and they were community women doing exactly what you said supporting each other.

Similarly, several support persons highlighted some of the same key points as the health care providers during group discussions. Many of the support persons mentioned the roles they played in providing emotional, informational, and financial support to the mother they care for. One father stated:

During her pregnancy, even now, I provide the emotional and mental support. Every now and then I support her financially if she really needs anything, whenever she needs someone to talk to. Just someone to keep her laughing as the day go out. Someone that she needs to talk to someone, she can lean, I’m that support.

It is evident emotional, informational, and financial support of the fathers, as well as health care providers, is vital to a mother’s well-being and her ability to care for a newborn. This reduces stress in the mother’s life and gives her the time needed to adjust to a new normal and care for herself and her body as she does so.

### Need for Technology to Access Information and Resources

Participants were shown a video about the PM^3^ mobile application and were asked to provide their perspectives to inform mobile application design to effectively assist Black, rural postpartum mothers. When support persons were also asked about the kinds of resources they would like to utilize through technology, one of them explained:

Have your resources of the app, be the go-to spot for mental health, for physical health, for financial health, these different resources that you can tie up with other websites where they can go to that website and find all this information. In addition to having the resources there, have a top—most asked questions where we will say like, how do I breastfeed? You have the information there for the mother. For the dads, what do I expect to happen with postpartum, have those questions.

Similarly, health care providers were asked about technology and how they currently utilize it during the postpartum period. For example, one mentioned:

I’ve developed a[n] educational module that I can submit to my patients through their health care portal. So, for my women who are way far out in rural Georgia, somewhere who are looking for prenatal care, and maybe can’t afford it, don’t have insurance, or whatever the reason may be, in addition to, to home visits, we do sort of its they’re like modules that are based on their weeks.

Alongside the many benefits of technology, health care providers were also asked to voice their concerns regarding any potential limitations for Black, rural postpartum mothers. One of them stated:

I think that people having a reliable phone, so I’m sure everyone has had the experience of patients who have a phone, and if they don’t have their minutes, then they can’t receive calls, they can’t text. So, there are some limitations and costs.

## Discussion

Our findings reveal the viewpoints of two key groups of individuals in a rural community on the support needs of rural postpartum Black mothers and how a mobile application could best support this population. The current study’s findings contribute to a better knowledge of the population’s, providers,’ and support persons’ concerns, as well as their identification of desirable app features that should be carefully considered in future mHealth interventions for rural women. During focus group discussions both HCPs and support persons mentioned the necessity of providing a community for mothers and highlighted this requires anyone in a support or provider role to be able to listen to mothers and provide what they need. The Sojourner Syndrome framework, used as the basis for this study, acknowledges the multifaceted roles and identities of Black women, as well as their capacity to cultivate resilience amid oppression. Factors such as race and rurality could contribute to the lack of support that mothers receive from providers and support persons in addition to the many other aspects of Black women’s identities that influence adverse health outcomes. For this reason, it would be necessary to leverage resources that provide a sense of support and community to mothers in the PM^3^ mobile application.

Feedback received on the PM^3^ mobile application suggested opportunities for a multidisciplinary and integrative experience for the mothers and their support persons, addressing both their physical and emotional needs. Moreover, a community forum to provide social support within the mobile application was suggested to be a pivotal part of the design of PM^3^.

### Strengths and Limitations

Although one of the main focuses on this article is rurality, 40% of the health care providers who participated in focus groups did not practice in a rural area. Recruitment, in general, posed a challenge, as it took place during the COVID-19 pandemic and there were many communication barriers with rural participants. For example, some participants lacked access to the necessary devices or networks to connect to Zoom sessions. Despite these challenges, thematic saturation was achieved, as we observed that several of the themes discussed during the focus groups were consistently reiterated.

In addition, there is limited existing literature on the impact of mHealth applications for Black, postpartum women living in rural areas; therefore, some of the references included in this article are older than ten years. This further highlights the need to examine the impact of mHealth interventions on rural, postpartum Black mothers.

## Conclusion

The information shared in the focus groups is valuable as it reports the direct experiences of obstetric health care providers and support persons to Black women in rural Georgia during the postpartum period. These findings are aligned with the target population of the PM^3^ mobile application and indicate the necessity for mHealth tools. Future research should evaluate the depth of social support channels and the outcomes of building sustainable connections with health care providers. Furthermore, the data collected in this study can foster safer relationships for mothers, their providers, and support persons and serve as a baseline for standardizing mHealth applications for Black mothers during the postpartum period. Moreover, while mHealth applications are growing in popularity, more studies are needed to ensure the usability and validity of the applications and examine their use in improving postpartum maternal health outcomes.

## Data Availability

The data sets used and/or analyzed during the current study are available from the corresponding author on reasonable request.

## Abbreviations Used

ARNP: advanced registered nurse practitioner
CAB: community advisory board
CNM: certified nurse midwife
HCP: healthcare provider mHealth: mobile health
MMM: Maternal Mortality/Morbidity Measurement Framework
PA: physician assistant
PM^3^: Prevent Maternal Mortality Using Mobile Technology
SRQR: Standards for Reporting Qualitative Research
WHO: World Health Organization
